# Prevalence of cannabis use disorders and associated factors among privately insured adults with epilepsy

**DOI:** 10.3389/fneur.2025.1695511

**Published:** 2025-12-01

**Authors:** Katarzyna Czerniak, Youngran Kim, Refugio Sepulveda, Sahiti Myneni, Robert Addy, Ross Shegog

**Affiliations:** 1 Center for Health Promotion and Prevention Research, School of Public Health, The University of Texas Health Science Center at Houston, Houston, TX, United States; 2 Department of Management, Policy and Community Health, School of Public Health, The University of Texas Health Science Center at Houston, Houston, TX, United States; 3 Mel and Enid Zuckerman College of Public Health, University of Arizona, Tucson, AZ, United States; 4 Department of Clinical and Health Informatics, D. Bradley McWilliams School of Biomedical Informatics, Houston, TX, United States

**Keywords:** substance use, epilepsy, cannabis, marijuana, substance use disorders

## Abstract

**Background:**

As cannabis is increasingly used as a treatment option for epilepsy, people with epilepsy (PWE) may be at higher risk of developing cannabis use disorder (CUD). This study aimed to estimate the prevalence of CUD among privately insured PWE in the United States using IQVIA’s 2022 PharMetrics® Plus for Academics health plan claims database.

**Methods:**

A cross-sectional analysis was conducted using 2022 IQVIA PharMetrics® Plus for Academics health plan claims data containing longitudinal view of inpatient and outpatient services, prescription and office/outpatient administered drugs, costs and detailed enrollment information. Individuals with epilepsy aged 18 years and older were included. ICD-10-CM codes were used to identify epilepsy, CUD, and comorbidity conditions including mood disorders, anxiety, tobacco use, alcohol use, migraines, and sleep apnea. The prevalence of CUD was estimated, and associations between CUD and potential predictors were examined using multivariable modified Poisson regression to calculate adjusted risk ratios (aRRs).

**Results:**

The prevalence of CUDs among U.S. PWE was 1.1% based on 2022 data from an analytic sample of 63,713 unique enrollees. PWE were on average 54.3 ± 17.2 years old and 53.6% were female. PWE with CUD were more likely to be young adult males and those 18–34 years old. PWE with CUD had six times more tobacco use and three times more mood disorders than those without CUD. PWE 18–24 years old were associated with a more than five times higher risk for CUD compared to PWE who were 65+ (aRR: 5.04, 95% CI: 3.83–6.65, *p*-value: < 0.001). Tabacco and alcohol use were associated with 6-fold increase (aRR: 5.8, 95% CI: 4.89–7.06, *p*-value: < 0.000) and 3-fold increase (aRR: 2.83, 95% CI: 2.21–3.64, *p*-value: < 0.001) respectively.

**Conclusion:**

PWE with substance use and psychiatric disorders are more likely to have CUD. Given the widespread prevalence of marijuana legalization across the U.S., increased awareness and potential screening for substance use disorders needs to be considered in epilepsy clinics.

## Highlights

- The prevalence of cannabis use disorders (CUDs) among U.S. patients with epilepsy in 2022 was estimated to be around 1.1%.
- Being younger in age, being male, and/or having a mood disorder or substance use disorder was significantly associated with an increased risk for CUD.
- Alcohol use was associated with three times increased risk of CUD despite previous studies stating that alcohol use disorders were associated with lower odds of CUD.
- Health care providers need to consider raising awareness of and screening for substance use disorders as well as providing cessation support in clinics with high-risk PWE.

## Introduction

An epilepsy diagnosis poses severe challenges because of uncontrolled seizures, reduced mental function, the complexity of treatment, social disadvantages (e.g., unemployment), stigma, and increased risk for comorbidities (i.e., anxiety and depression) and early mortality (1–3). The use of cannabis, also known as marijuana, as an anticonvulsant has been reported to suppress seizures, but evidence is mixed. The 2018 National Academies of Sciences, Engineering, and Medicine (NASEM) report reviewed two systematic reviews assessing the effects of cannabis for reducing seizures in patients with epilepsy and found insufficient evidence to support a therapeutic effect (4). Other more recent studies have found that cannabidiol (CBD), one of many cannabinoids found in marijuana, has demonstrated antiseizure activity in well-designed randomized placebo-controlled trials (5, 6). The effect of cannabis on seizures has been found to be inconsistent in general and yet a 2019 cross-sectional study analyzing 337 Canadian patients with epilepsy found that 21% of these PWE reported cannabis use (7). According to the National Survey on Drug Use and Mental Health, marijuana was the third most commonly used drug in the United States in 2023, with 15.4% of people aged 12 or older (or 52.5 million people) having reported using it in the past month, right behind tobacco use (17.6%) (8).

Cannabis is recognized as a harmful substance due to risk of aggravating psychosis, reduction of cognitive functions leading to traffic accidents, its psychosocial consequences, and the adverse physical effects it has on pregnancy and the respiratory system (9). Of most concern is that higher frequencies of cannabis use have the potential to lead to dependence and abuse collectively referred to as cannabis use disorder. The CDC reports that approximately 3 in every 10 marijuana users develop a cannabis use disorder (10) which is specifically defined as a disorder that occurs when constant marijuana use leads to physical and social issues and the user begins experiencing mental and physical difficulties with quitting (11). A cross-sectional study reported that adult epilepsy patients with a cannabis use disorder (CUD) were found to have threefold higher odds of epilepsy emergency admissions (OR: 3.36; 95% CI: 3.22–3.50; *p* < 0.001) compared to epilepsy patients without CUD (12). In addition, higher frequency cannabis use is associated with younger age (ages 18–34 years: adjusted odds ratio [aOR]: 4.12; 95% CI: 3.63–4.68; ages 35–64 years: aOR: 2.22; 95% CI: 1.98–2.49) (13) which is associated with a more rapid transition to substance use disorders (SUDs) and higher rates of psychiatric disorders (14).

Individuals with epilepsy are also twice as likely to have a psychiatric diagnosis compared to those without epilepsy (15). Depression is the most frequent psychiatric comorbidity in epilepsy affecting over 23% of people with epilepsy in community-based studies and is a well-known predictor of substance use (16). In general, about 50% of patients with epilepsy have one or more comorbidities which are often associated with increased risk of substance use (17). As such, people with epilepsy have an almost 3 times increased odds for a substance use disorder than those without epilepsy (OR: 2.75; 95% CI: 1.61–4.72) (15). Several diseases, including anxiety, dementia, and migraines are also up to eight times more common in people with epilepsy than in the general population (18).

Despite previous estimates, the prevalence and incidence of CUD among people with epilepsy is difficult to ascertain due to the rarity of such studies and the limitations associated with them. Most studies in this area utilized self-reporting within national surveys to determine marijuana use frequency. However, there were two studies that utilized administrative healthcare data to analyze validated diagnoses of CUD. A 2019 cross-national analysis of 2010–2014 data taken from the United States Nationwide Inpatient Sample (NIS) database reported the incidence of CUD to be 5.77% in a sample of over 650,000 hospitalized patients (12) while a 2020 study that analyzed 2006–2014 NIS data found CUD prevalence increased among epilepsy patients (2.18% in 2006–4.41% in 2014) (19). The NIS contains data on around 35 million hospitalizations in the U.S. but the exclusive use of hospitalized patient data limits generalizability to all epilepsy patients. National-level administrative healthcare datasets, however, allow for the chance to estimate epilepsy and CUD prevalence using validated algorithms with data representative of both inpatient and outpatient settings (20). As such, the purpose of this study is to estimate the period prevalence of cannabis use disorders over 2022 among adult patients with epilepsy in the United States by analyzing claims data from IQVIA’s national payer database, the IQVIA PharMetrics® Plus for Academics health plan claims database.

## Methods

### Study design and dataset

A retrospective cross-sectional analysis of claims data was conducted using 2022 data from the IQVIA PharMetrics® Plus for Academics health plan claims database (21). IQVIA is an independent third-party company that collects adjudicated or pre-adjudicated claims captured from across all settings of care from health insurers and healthcare providers. The dataset contains data on over 200 million enrollees across the United States and is comprised of fully adjudicated medical and pharmacy claims providing a comprehensive view of inpatient and outpatient services, prescription and office/outpatient administered drugs, costs and enrollment information.

### Study participants

The operational definition of a case of epilepsy for this study was based on the International Classification of Diseases, Tenth Revision, Clinical Modification (ICD-10-CM) coded diagnoses of epilepsy or seizures which is the primary standardized classification system used to code clinical diagnoses in the United States for billing purposes (22). Cases of epilepsy were identified as an occurrence of at least one ICD-10-CM epilepsy code (G40. XX) for any diagnosis field among any medical claims among those at least 18 years of age or older enrolled between January 1, 2022 and December 31, 2022. Those younger than 18 years old with a diagnosis of pediatric-related epilepsy were excluded as this study aims to focus on adult epilepsy.

The CDC defines an “active epilepsy” case as having a history of doctor-diagnosed epilepsy and taking medication to control it, having one or more seizures in the past year, or both (23). A systematic review conducted to ascertain the accuracy of using administrative healthcare data to identify epilepsy cases identified 28 studies which generated 172 algorithms estimating PPV. 121 of these algorithms (generated by 13 studies) also estimated sensitivity. The results indicated that claims data can accurately identify epilepsy cases, with optimal estimates of positive predictive value (PPV), sensitivity, negative predictive value (NPV), and specificity >80% in the majority of studies (20). Algorithms using only diagnostic codes managed to retain both PPV and sensitivity >80% indicating that diagnostic codes alone are sufficient so neither drug codes nor symptom codes were used to identify epilepsy cases (20). Since ICD-10-CM epilepsy codes have been repeatedly validated in previous studies, no additional validation analyses were conducted as part of this study.

### Cannabis use disorder case identification

Prevalent cases of cannabis use disorder were identified from among patients with epilepsy within the denominator population who meet the operational definition of a case of cannabis use disorder. Cases were identified as an occurrence of at least one ICD-10-CM cannabis use disorder code (F12.1XX, F12.2XX, or F12.9XX) for any diagnosis among any medical encounter. The ICD-10-CM codes for both cannabis abuse (F12.1XX) and dependence (F12.2XX) were used for this study as part of the definition of cannabis use disorder as they aligned with previous studies using administrative healthcare data (12, 19). In addition, both are classified under the term cannabis use disorder in the Diagnostic and Statistical Manual of Mental Disorders, Fifth Edition, Text Revision (DSM-V-TR) which is the standard used in the United States to help clinicians and researchers define and classify mental disorders (24).

### Demographics

Demographic factors that were assessed were age, gender, and region. Age was calculated by subtracting the year of birth from the date of service. However, those 85 and older had their year of birth programmatically set to 0000 in the data and so were dropped from the analysis since this made it impossible to calculate their age. Gender was listed as M for Male, F for Female, or U for Unknown. Patients who had “Unknown” gender were also dropped as this indicated that they chose not to provide their gender during enrollment. For region, patients were assigned to one of four U.S. Census regions based on residence: E for Northeast, S for South, MW for Midwest, and W for West. Those whose region of residence was unknown were assigned O and were subsequently dropped from the analysis.

U.S. State of residence data was available for each patient, but the final sample was too limited to conduct a state-wide analysis and the large number of states led to relatively small sample sizes. In addition, there are a variety of cannabis policies in place to control access to the drug, and because they differ between states this made it difficult to obtain any meaningful comparisons. As such, CUD prevalence was stratified by U.S. region only.

### Associated factors/covariates

Comorbid conditions commonly observed among PWE were included when assessing the prevalence of CUD in this population. These covariates included mood disorders, anxiety, tobacco use, alcohol use, sleep apnea and migraine. Cases for each covariate were identified as an occurrence of ≥1 ICD-10-CM related code(s) for any diagnosis among any medical encounter. The ICD-10-CM diagnosis codes and validated algorithms for each covariate are described in Table 1.

**Table 1:** Validated ICD-10-CM algorithms for covariates used in final analysis.

Covariates	ICD-10-CM codes	Validation	Source
Mood disorders (depression and/or bipolar)	F30. XX, F31. XX, F32. X, F33. XX, F34XX	PPV = 89.7% (depression) and 89.5% (bipolar); often used together	Fiest et al. (26)
Anxiety	F40. X, F41. X	Sensitivity = 76.2%	Howren et al. (28)
Tobacco use	F17.2X, Z72.0X	PPV = 81.5%	Haque et al. (29)
Alcohol use	F10.1X, F10.2X, F10.9X, Z71.4X	PPV = 87.4% Specificity = 96.9% NPV = 90.8%	Kim et al. (30)
Migraine	G43. XXX	PPV = 81.7% Specificity = 99.2% NPV = 87.5%	Kolodner et al. (31)
Sleep apnea	G47. XX	PPV = 89.1% Sensitivity = 79.2%	Jolley et al. (32)

*Mood disorders:* Depression and bipolar disorders were combined into a single variable and treated as mood disorders due to high comorbidity between the two. Due to the many similarities in symptomology between depression and bipolar disorders, the two are commonly misdiagnosed and quite often diagnosed together (25). In addition, a systematic review and assessment of validated case definitions for depression in administrative data found that ICD-9 and ICD-10 case definitions used both depression and bipolar codes together (26).

*Anxiety:* Only a handful of studies exist that use anxiety diagnostic codes within administrative healthcare data as anxiety is difficult to diagnosis accurately due to its high comorbidity with most psychiatric and chronic clinical conditions including patients with epilepsy (27). A systematic review that looked for validation studies for depression and anxiety diagnostic codes among individuals with rheumatic diseases found a single validation study for anxiety ICD codes (28).

*Tobacco use:* A systematic review that assessed the validity of measuring tobacco status using administrative healthcare data found that algorithms based on EMR data had a higher median PPV than those based on administrative data (91% vs. 81.5%) (29). Since accessing EMR data is beyond the scope of this study and PPVs remained >80%, only diagnostic codes were used to identify tobacco use.

*Alcohol use:* ICD codes for alcohol use were validated in a study that looked at Veterans Health Administration claims data using three random samples of patients. In the combined sample, PPV of alcohol dependence and abuse diagnosis in administrative data was 87.4%, specificity was 96.9%, and NPV was 90.8% (30).

*Physical comorbidities:* There are several physical comorbidities that are common among PWE which include apnea (sleep disorder) and migraine. ICD codes were validated in previous research studies which reported a PPV of 89.1% for sleep apnea and 81.7% for migraine (31, 32).

### Data analysis

Descriptive statistics were calculated using frequency and means. Overall prevalence was calculated by dividing the total number of CUD cases among people with epilepsy (PWE) in 2022 by the total number of PWE enrolled in the 2022 calendar year then stratified by gender, sex, and region. Modified Poisson regressions were run to assess the association between CUD and characteristics among this population. Crude risk ratios were calculated to account for any possible over dispersion. Bivariate modified Poisson regressions were conducted to assess the association between factors and comorbidities that are significantly associated with CUD among PWE followed by a multivariable modified Poisson regression analysis (33). Significance levels were set at *p* < 0.05 for 2-tailed tests, and all analyses were performed using STATA 18.0 (StataCorp) (34).

## Results

### Characteristics of study participants

**Figure 1:**
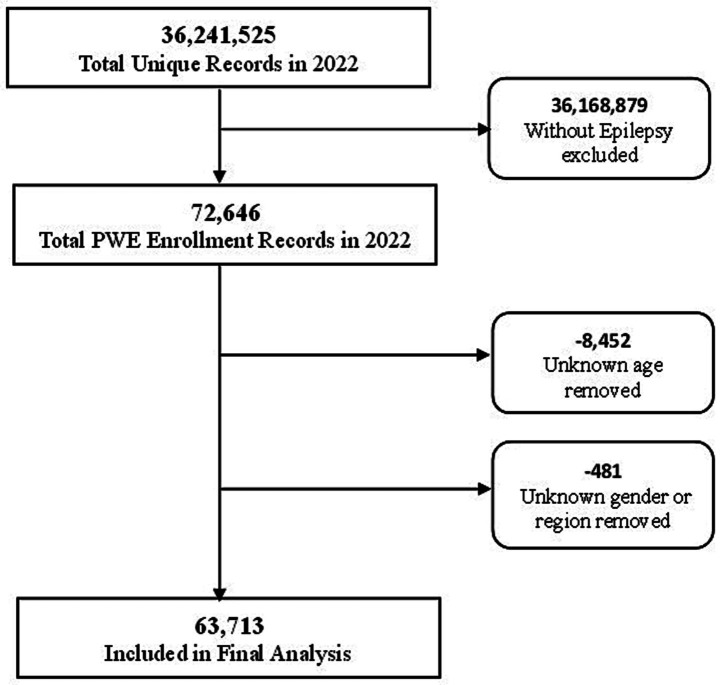
Flow chart of sample selection. The figure shows how the final sample size was derived from the 32,241,525 unique patient cases enrolled in 2022. Inclusion/exclusion criteria was determined using demographic information and ICD-10-CM diagnosis codes.

From the 2022 IQVIA data, 63,713 unique enrollees met the criteria for a diagnosis of epilepsy and were included in the final analysis as shown in Figure 1. Of those, there were 699 (1.1%) patients with a diagnosis of epilepsy who also had a cannabis use disorder (CUD) diagnosis. As seen in Table 2, PWE were on average 54.3 ± 17.2 years old and 53.6% were female (Table 2). PWE with CUD were much younger (mean age 44.3 vs. 54.4 years) and more likely to be male compared to those without CUD (60.9% vs. 46.2%). There were no major differences between groups when comparing frequencies across region. The majority of study participants resided in the Midwest region of the United States followed by the South region.

**Table 2:** Characteristics of commercially insured PWE in the United States in 2022.

Variable	Total PWE (*N* = 63,713)	PWE with CUD (*N* = 699)	PWE without CUD (*N* = 63, 014)	*p*-value
Age (mean, SD)	54.3 ± 17.2	44.3 ± 16.01	54.4 ± 17.2	*p* < 0.001
Age category (*n*, %)				
18–24	4,259 (6.7)	90 (12.9)	4,169 (6.6)	*p* < 0.001
25–34	7,024 (11.02)	138 (19.7)	6,886 (10.9)	
35–44	7,474 (11.8)	141 (20.2)	7,333 (11.6)	
45–54	9,192 (14.4)	108 (15.4)	9,084 (14.4)	
55–64	12,850 (20.2)	120 (17.2)	12,730 (20.2)	
65+	22,914 (35.9)	102 (14.6)	22,812 (36.2)	
Gender (*n*, %)				
Male	29,541 (46.4)	426 (60.9)	29,115 (46.2)	*p* < 0.001
Female	34,172 (53.6)	273 (39.1)	33,899 (53.8)	
Region (*n*, %)				
Northeast	9,020 (14.2)	89 (12.7)	8,931 (14.2)	0.76
South	18,949 (29.4)	199 (28.5)	18,750 (29.8)	
Midwest	23,922 (37.5)	281 (40.2)	23,641 (37.5)	
West	11,822 (18.5)	130 (18.6)	11,692 (18.5)	
Comorbidities (*n*, %)				
Mood disorder (depression and/or bipolar)	9,487 (14.9)	283 (40.5)	9,204 (14.6)	*p* < 0.001
Anxiety	8,342 (13.1)	210 (30.0)	8,132 (12.9)	*p* < 0.001
Tobacco use	3,878 (6.1)	262 (37.5)	3,616 (5.7)	*p* < 0.001
Alcohol use	1,086 (1.7)	84 (12.02)	1,002 (1.6)	*p* < 0.001
Migraine	4,571 (7.2)	50 (7.2)	4,521 (7.2)	0.25
Sleep apnea	5,989 (9.4)	83 (11.9)	5,906 (9.4)	0.007

Mood disorders were the most frequent conditions among PWE followed by anxiety. Among PWE with CUD, mood disorders (40.5% vs. 14.6%) and anxiety (30.0% vs. 12.9%) were also significantly more prevalent compared to those without CUD. Tobacco use prevalence, however, was approximately 6.5 times higher among PWE with a CUD compared to PWE without CUD (37.5% vs. 5.7%) and alcohol use was approximately seven times higher (12% vs. 1.6%). There were no observed significant differences in prevalence of migraine and sleep apnea frequencies between PWE with and without CUD (Table 2).

Poisson bivariate analyses, as seen in Table 3, revealed that age and gender were both significantly associated with CUD risk among PWE (Table 3). PWE between the ages of 18 and 44 were associated with an over four times higher risk for CUD compared to older PWE over the age of 65 (RRs: 4.2–4.7, *p*-values: < 0.001). In addition, male PWE were associated with an almost two times higher risk for CUD compared to females (RR: 1.8, 95% CI: 1.5–2.1). The U.S. region that PWE lived in was not significantly associated with CUD (*p*-values: 0.1–0.6).

**Table 3:** Modified Poisson regression of factors associated with CUD among PWE in the US in 2022.

Variable	CUD prevalence	Bivariate poisson regression		Multiple poisson regression	
		Crude RR [95% CI]	*P*-value	Adjusted RR [95% CI]	*P*-value
Age category, *n* (%)					
18–24	2.1%	4.7 [3.5–6.3]	<0.001	5.0 [3.83–6.65]	<0.001
25–34	1.9%	4.4 [3.4–5.7]	<0.001	3.9 [3.05–5.07]	<0.001
35–44	1.8%	4.2 [3.3–5.5]	<0.001	3.2 [2.51–4.18]	<0.001
45–54	1.2%	2.6 [2.0–3.4]	<0.001	1.8 [1.42–2.45]	<0.001
55–64	0.93%	2.1 [1.6–2.8]	<0.001	1.5 [1.21–2.05]	0.001
65+	0.44%	1.00 (reference)		1.00 (reference)	
Gender, *n* (%)					
Male	1.4%	1.8 [1.5–2.1]	<0.001	1.74 [1.49–2.02]	<0.001
Female	0.79%	1.00 (reference)		1.00 (reference)	
Region, *n* (%)					
South	1.0%	1.1 [0.83–1.4]	0.60	–	–
Midwest	1.2%	1.2 [0.94–1.5]	0.10	–	–
West	1.1%	1.1 [0.85–1.4]	0.40	–	–
Northeast	0.98%	1.00 (reference)		–	–
Comorbidities, *n* (%)					
Mood disorder (Depression/Bipolar)					
Yes	2.9%	3.9 [3.3–4.5]	<0.001	2.65 [2.23–3.14]	<0.001
No	0.76%	1.00 (reference)		1.00 (reference)	
Anxiety					
Yes	2.5%	2.8 [2.4–3.3]	<0.001	1.47 [1.23–1.75]	<0.001
No	0.88%	1.00 (reference)		1.00 (reference)	
Tobacco use					
Yes	6.7%	9.2 [7.9–10.7]	<0.001	5.88 [4.89–7.06]	<0.001
No	0.73%	1.00 (reference)		1.00 (reference)	
Alcohol use					
Yes	7.7%	7.9 [6.3–9.8]	<0.001	2.83 [2.21–3.64]	<0.001
No	0.98%	1.00 (reference)		1.00 (reference)	
Migraine					
Yes	1.1%	0.9 [0.74–1.3]	0.98	–	–
No	1.1%	1.00 (reference)		–	–
Sleep apnea					
Yes	1.4%	1.3 [1.03–1.6]	0.02	0.99 [0.79–1.25]	0.97
No	1.1%	1.00 (reference)		1.00 (reference)	

Substance use disorders were associated with the highest risk of developing CUD among PWE. Tobacco using PWE were associated with a nine times higher risk for CUD (aRR: 9.2, 95% CI: 7.9–10.7) in comparison to non-tobacco using PWE, and alcohol use was associated with an almost eight times higher risk (aRR: 7.9, 95% CI: 6.3–9.8). Mood and anxiety disorders were also significantly associated with increased risk for CUD. Having a mood disorder was associated with a four times higher risk for CUD among PWE (aRR: 3.9, 95% CI: 3.3–4.5) than for those without while anxiety disorders were associated with an almost three time higher CUD risk (aRR: 2.8, 95% CI: 2.4–3.3). PWE with sleep apnea were associated with an almost 1.5 times higher risk for CUD than PWE with no sleep apnea (aRR: 1.3, 95% CI: 1.03–1.6). Migraine was the only covariate that had no statistically significant association with CUD risk.

### Multivariable analysis of CUDs among patients with epilepsy

To assess the independent associations between CUD and potential factors, a multivariable modified Poisson regression, as shown in Table 3, was run which included age, gender, mood disorder, anxiety, tobacco use, alcohol use and sleep apnea (Table 3). Factors with non-significant *p*-values of > 0.05 at the bivariate level were not included. Goodness-of-fit tests performed *ad hoc* showed that the multivariable model fit well (*p*-value = 1.000). When controlling for all significant factors and covariates, the multivariable model demonstrated decreasing prevalence of CUD by age. PWE who were 18–24 years old were associated with a five times greater risk for CUD then PWE who were 65 and older (aRR: 5.04, 95% CI: 3.83–6.65) while the association with gender was retained. Among the covariates, tobacco use was associated with the highest risk of developing a CUD among PWE but the risk ratio of 9 fell to almost 6 when adjusting for all other variables (aRR: 5.8, 95% CI: 4.89–7.06). Alcohol use and anxiety disorders were still significantly associated with CUD risk among PWE but again the adjusted ratios fell in the multivariable analysis to 2.8 and 1.5, respectively. Sleep apnea did not retain statistical significance in the multi variable model.

## Discussion

The purpose of this cross-sectional study was to estimate the prevalence of cannabis use disorder and identify its associated covariates among adult patients with epilepsy in the United States using administrative data extracted from the 2022 national payer IQVIA PharMetrics® Plus claims database. The prevalence of CUDs among U.S. patients with epilepsy was estimated to be approximately 1.1%. The prevalence of CUD was lower than that reported in previous research. Patel et al. reported an overall CUD prevalence of 5.77% among PWE when analyzing NIS data from 2010 to 2014 and Lekoubou et al. reported that 4.41% of PWE from the NIS database had a CUD in 2014 (12, 19). The most likely explanation for this is that NIS contains data only from hospitalized patients whereas IQVIA contains claims from both inpatient and outpatient settings. Cannabis use in general is associated with higher odds of hospitalization which indicates that CUDs are more likely to be found among hospitalized patients rather than healthier outpatients (12).

Further, IQVIA is a commercial database that contains claims from patients with privately owned insurance, which may reflect greater access to healthcare, higher SES, and related elevated quality of life compared to PWE with public or government insurance like Medicare (35). This may have possibly resulted in a lower prevalence of epilepsy cases since socioeconomic deprivation is known to increase the incidence and prevalence of epilepsy due to the fact that PWE in general have lower education and more difficulty finding work than the healthy population (36). However, non-insured and publicly insured PWE have significant gaps in access to specialized epilepsy services. Due to lack of coverage, they are less likely to seek treatment at epilepsy clinics and use antiseizure medication (36). Future studies combining commercial claims data sets with claims data focused on the publicly insured (such as Medicare) may potentially address these gaps providing prevalence rates that are more generalizable to the broader PWE population.

Previous research indicates that within the general population, people with CUD are two times more likely to have another psychiatric disorder and experience poorer wellbeing (37). PWE on the other hand are already at an increased risk of psychiatric comorbidities due to underlying neurobiological mechanisms and psychosocial factors that closely link epilepsy with poorer mental health and reduced quality of life (38). As such, initiating cannabis use compounds these risks greatly among PWE who are much more likely to self-treat than the general population due to existing inconsistent information on the anticonvulsant properties of cannabis. This is consistent with the study findings that PWE with CUD were associated with a six times greater likelihood of having an additional psychiatric diagnosis, double that of the general population. These findings have implications for epilepsy treatment and self-management as psychiatric comorbidities are a known predictor of lower quality of life and impede PWEs’ ability to manage their condition.

Being younger in age and male were associated with an increased risk of CUD. Having mood disorders, anxiety, tobacco use, or alcohol use also significantly increased the risk of CUD. These findings are consistent with previous research using the National Impatient Sample (NIS) database which found that CUD was more likely to be present in young male epilepsy patients with depression, bipolar disorder, and tobacco use disorder (19). However, Lekoubou et al. (19) found alcohol use disorders to be associated with lower odds of CUD. In contrast, alcohol use within this study was associated with an almost three times higher risk of CUD. This discrepancy is highly unusual as alcohol use disorders have been found to have strong positive associations with CUD even among the general population (39). An earlier study that analyzed associations between substance use disorders and hospitalizations among PWE found that alcohol use disorders were associated with higher odds of epilepsy hospitalization (12). However, this study did not examine the associations between alcohol and CUD (12), and a more recent study that used NIS data to assess CUD prevalence among epilepsy patients did not assess substance use comorbidities (12, 40). There is a possibility that the models presented were impacted by extraneous factors resulting in misleading information (40). Further, implementation of recreational cannabis policies has resulted in increased simultaneous use of cannabis and alcohol among the general population as well as patients who use these substances medically (41, 42). As such, additional longitudinal studies utilizing claims data from both publicly and privately insured PWE are needed to ascertain the current relationships between CUD and alcohol use disorders among this population.

There is a growing body of evidence that indicates that substance use may negatively impact seizure frequency. As such, health care providers need to consider raising awareness of and screening for substance use disorders as well as providing cessation support in clinics with high-risk PWE. Evidence-based cessation resources integrated into epilepsy self-management programs may also help reach high-risk PWE outside the clinic setting.

### Limitations and strengths

There are several potential limitations regarding the use of claims data to estimate CUD prevalence. First, the IQVIA PharMetrics® Plus for Academics database contains claims from commercially insured individuals only so findings may not be generalizable to individuals who are uninsured, or covered by Medicare or Medicaid. Secondly, since a health care encounter is needed to generate claims data those choosing not to be treated for their epilepsy and those who have “inactive” epilepsy that does not require treatment were also not captured in the data. In addition, the IQVIA Pharmetrics dataset does not provide information on detailed sociodemographic characteristics such as ethnicity and race which limits comparison analyses to gender and age only.

Further, tobacco use is known for being underreported in administrative claims data and algorithms that rely only on claims data typically have limited sensitivity but very high specificity. This means that prevalence rates and risk ratios in this study may potentially be underestimated. A systemic review identified 116 algorithms used to identify tobacco cases and almost three-quarters were based on EMR data. In addition, algorithms for EMR data were reported to have higher sensitivity than those for claims data. Future study designs are needed that integrates EMR data with claims data to accurately validate smoking status and address this limitation.

Finally, the cross sectional study design limits interpretation to associations since data was collected from only a single time period. As such, causality between CUD, epilepsy, and associated co-morbidities could not be established. Follow-up longitudinal analyses are needed to properly assess temporal relationships between CUD and its associated factors. Despite these limitations, previous CUD prevalence studies among PWE have been conducted but such studies looked only at hospitalized patients or veterans. Strengths of this research are the inclusion of both inpatient and outpatient claims data enabling broader examination beyond hospitalized and elderly populations as well as the use of validated CUD diagnoses rather than self-reported surveys.

## Conclusion

This cross-sectional analysis examines the prevalence of CUDs among PWE enrolled in a national commercial claims database containing both inpatient and outpatient data. The period prevalence of CUDs among U.S. patients with epilepsy in 2022 was approximately 1.1%. CUD in PWE was strongly associated with a higher risk for other substance use disorders. PWE with CUD were six times more likely to use tobacco and three times more likely to use alcohol than PWE with no CUD. With legalization of cannabis consistently spreading across the United States, PWE now have increased access, suggesting an increased need to screen for CUD and other substance use disorders in high-risk epilepsy patients. The Diagnostic and Statistical Manual of Mental Disorders, Fifth Edition (DSM-V) lists 11 symptoms that can be used to diagnose CUD if a person exhibits at least two of these symptoms within a 12-month period. The Cannabis Use Disorders Identification Test-Revised (CUDIT-R) utilizes 8 self-report items based on these DSM-V criteria and is the most widely used instrument for assessing hazardous cannabis-use. It is a generally recognized and psychometrically sound instrument for identifying CUD in general clinical and young adult populations (43). In addition, evidence suggests that cognitive-behavioral therapy with motivation enhancement, and dialectical behavioral/acceptance and commitment therapy may increase abstinence among people with cannabis use disorder (44). The findings suggest that there is a need for integrated care in epilepsy management utilizing screening tools, cessation support, and psychosocial services tailored to high-risk PWE. The results of this study should be used to inform evidence-based epilepsy programs and the decisions of public health practitioners, health care providers, policy makers, researchers, and other stakeholders.

## Data Availability

The data analyzed in this study is subject to the following licenses/restrictions: the data that support the findings of this study are third-party administrative claims data. Restrictions apply to the availability of the claims data, which were used under license for the current study, and so are not publicly available. Requests to access these datasets should be directed to IQVIA at https://www.iqvia.com/locations/united-states/library/fact-sheets/iqvia-pharmetrics-plus-for-academics.
